# Editorial: Characterising the functional heterogeneity of synovial macrophages

**DOI:** 10.3389/fimmu.2026.1907859

**Published:** 2026-06-30

**Authors:** Anna B. Montgomery, Deborah R. Winter

**Affiliations:** 1 Centre for Inflammation Biology and Cancer Immunology (CIBCI), King's College London, London, United Kingdom; 2 Feinberg School of Medicine, Northwestern University, Chicago, IL, United States

**Keywords:** macrophage biology, myeloid biology, osteoarthritis, rheumatoid arthritis, synovium

Macrophages are essential homeostatic cells in all human tissues, and one of the first to form during embryogenesis. In collaboration with their supporting tissue niche, macrophages perform essential homeostatic functions specific to tissue location (1), from recycling iron in the liver to pruning synapses in the brain (2). In contrast, macrophages are recruited to tissues in response to inflammatory cues through differentiation of peripheral blood monocytes where they perform inflammatory and cytotoxic functions in response to infection (3). This dichotomy of functions is exhibited clearly in the joint synovium, where anatomical location in the synovial lining, sublining, and perivascular compartments is tightly connected to function (4). In this Research Topic entitled *“Characterizing the Functional Heterogeneity of Synovial Macrophages”* authors advance upon this understanding to resolve further complexity, and examine how distinct macrophage subsets contribute to joint homeostasis, inflammation, aging, fibrosis, and arthritis. Collectively, the papers emphasize that synovial macrophages exhibit divergent pathogenic or protective functions, driven by a range of local and systemic signals.

In this special edition, a mini-review on the ontogeny of synovial macrophages, elegantly synthesised the current understanding of macrophage developmental origins, and how this impacts synovial macrophage function. The authors describe the change in the synovial macrophage niche that accompanies inflammation referencing studies using both mouse models and patient samples. By addressing the question of ‘does origin matter?’ Hutton et al. evaluate how macrophage subpopulations contribute to homeostasis and pathology (5).

Building on previously published works using bone marrow chimeras and transgenic mice to resolve heterogeneity in the synovial macrophage compartment (6, 7). Dapas et al. interrogate the effect of age and sex on the transcriptional profiles of synovial macrophages (8). This work is essential to address both the age-associated risks and sex bias of inflammatory joint disorders. The risk of developing rheumatoid arthritis (RA) doubles over the age of 60 (9), and according to the world health organization, over 70% of people living with osteoarthritis (OA) are older than 55 years. Furthermore, women are also disproportionately affected by both RA and OA (10, 11). To that end, Dapas et al. used single-cell RNA-seq (scRNA-seq) on isolated synovial macrophages from both male and female young and aged mice (8). Surprisingly, they observed a polarity in aging-induced transcriptional changes in males and females, suggesting sex-dependent remodelling of the synovial immune compartment. In aging females, a subset of Arg1-expressing MHCII^+^ macrophages were increased, in contrast to a decrease in aging males. Interestingly, Arg1 has previously been reported to play a protective role in age-related cartilage repair, and resolution of joint inflammation (12, 13), however both studies reported use of male mice only and so the effect in female mice remains elusive. In both sexes, Ly6c^+^ infiltrating macrophages were expanded while tissue-resident macrophages including CX3CR1^+^ lining and RELMα^+^ (*Retnla*^+^) interstitial macrophages were reduced, indicating ageing drives both shared and sex-specific changes. Based on these data, the authors conclude impaired macrophage differentiation and altered inflammatory signalling may contribute to age-associated arthritis susceptibility, highlighting the importance of studying sex-bias as a mechanism in inflammatory disease.

Schonfeldova et al. expanded further on synovial RELMα^+^ macrophages, investigating a central theme across the Research Topic; the importance of tissue localisation in synovial macrophage programming (14). The authors demonstrate that RELMα^+^ interstitial macrophages are a specialized perivascular population positioned near blood vessels in the synovium. From this location, RELMα^+^ interstitial macrophages act as a primary source of CCL2 to orchestrate recruitment of CCR2^+^ monocytes into the joint during inflammatory arthritis. Recruited monocytes subsequently differentiate into tissue macrophages, including synovial lining macrophages. These data suggest monocyte-derived macrophages can adopt a protective phenotype in synovial inflammation, contrary to current understanding. In addition, this work also supports previous reports highlighting the importance of vascular associated monocytes and fibroblasts in controlling synovial inflammation (15, 16).

Mechanisms of cell death is an understudied area of research in arthritis, with the ability to exacerbate inflammation. Pyroptosis is a form of inflammatory programmed cell death that has been implicated in the pathogenesis of OA (17, 18), however the relevance to specific cell types was previously unknown. In their original research article, Yao et al. performed a meta-analysis of multiple RNA-seq datasets from OA patients and controls to examine the expression of pyroptosis-related genes (19). Using a network approach and single-cell RNA-seq data, they identified PYCARD as a core pyroptosis gene in synovial macrophages associated with inflammation. The authors propose that the expression of the pyroptosis pathways has implications for therapeutic targeting.

Finally, the Research Topic includes a broader review on macrophage-myofibroblast transformation in fibrotic disease (20). Generalising beyond the joint, this paper explores how macrophages can transition toward myofibroblast-like phenotypes and contribute to fibrosis, extracellular matrix remodelling, and chronic tissue pathology. Within the context of synovial disease, this concept proposes a role for synovial macrophages in pathology of fibrotic synovitis, as well as inflammatory joint pathology.

Taken together, the Research Topic advances upon previous detailed characterisation to add functional relevance to synovial macrophage subpopulations. The combined collection emphasises that identity is shaped by origin, as well as local stromal and vascular niche, and influenced by age and sex. These influences impact the contribution of synovial macrophages to both homeostatic and damage-associated responses in synovial tissue, as well as in driving the recruitment of other immune cell populations. This collection of work raises exciting potential for macrophage targeting therapeutics as disease-specific and subpopulations contributions to joint pathology are further elucidated.
